# Efficacy and Safety of a Chinese Herbal Medicine Formula (RCM-104) in the Management of Simple Obesity: A Randomized, Placebo-Controlled Clinical Trial

**DOI:** 10.1155/2012/435702

**Published:** 2012-02-20

**Authors:** George Binh Lenon, Kang Xiao Li, Yung-Hsien Chang, Angela Weihong Yang, Clifford Da Costa, Chun Guang Li, Marc Cohen, Neil Mann, Charlie C. L. Xue

**Affiliations:** ^1^Traditional & Complementary Medicine Research Program, Health Innovations Research Institute, School of Health Sciences, RMIT University, Bundoora, VIC 3083, Australia; ^2^China Medical University and Hospital, Taichung City 40447, Taiwan; ^3^School of Mathematics & Geospatial Sciences, RMIT University, Bundoora, VIC 3083, Australia; ^4^School of Applied Sciences, RMIT University, Bundoora, VIC 3083, Australia; ^5^Guangdong Provincial Academy of Chinese Medical Sciences, Guangzhou, China

## Abstract

*Objective.* This study was to evaluate the efficacy and safety of a Chinese herbal medicine formula (RCM-104) for the management of simple obesity. *Method.* Obese subjects aged between 18 and 60 years were selected for 12-week, double-blind, randomized, placebo-controlled trial. Subjects were randomly assigned to take 4 capsules of either the RCM-104 formula (*n* = 59) or placebo (*n* = 58), 3 times daily for 12 weeks. Measures of BW, BMI and WC, HC, WHR and BF composition were assessed at baseline and once every four weeks during the 12 week treatment period. *Results*. Of the 117 subjects randomised, 92 were included in the ITT analysis. The weight, BMI and BF in RCM-104 group were reduced by 1.5 kg, 0.6 kg/m^2^ and 0.9% and those in the placebo group were increased by 0.5 kg, 0.2 kg/m^2^ and 0.1% respectively. There were significant differences in BW and BMI (*P* < 0.05) between the two groups. Eleven items of the WLQOQ were significantly improved in the RCM-104 group while only 2 items were significantly improved in the placebo group. Adverse events were minor in both groups. *Conclusion*. RCM-104 treatment appears to be well tolerated and beneficial in reducing BW and BMI in obese subjects.

## 1. Introduction

Obesity is a common metabolic disorder in developed and developing countries [1] and is characterized by weight gain, fatigue, and lassitude. Obesity is associated with serious health conditions such as hypertension (32.1%), osteoarthritis (38.9%), joint pain (27.4%), chronic insomnia (23.4%), allergy (37.3%), and depression (23.4%) [2]. The increasing prevalence of obesity is a major public health concern since obesity is often associated with cerebrovascular and cardiovascular diseases such as hypertension and arteriosclerosis, as well as diabetes mellitus and the acceleration of the aging process [3]. The prevalence of obesity has risen to alarming levels worldwide with the World Health Organization (WHO) estimating that there will be 2.3 billion overweight and 700 million obese adults by 2015. In Australia, 25% of the population is obese [4], whereas in the US it is estimated that 30.4% of adults are obese [5, 6]. 

Current management of obesity by pharmacotherapy includes noncentrally acting antiobesity agents such as Orlistat (Xenical), which inhibits the action of the intestinal lipase enzymes and hence blocks the absorption of fat in the intestines. The most common adverse events of Orlistat include oily faecal spotting, abdominal pain, and flatus with discharge, faecal urgency, fatty/oily stool, increased defecation, and faecal incontinence [7].

Another pharmacotherapy is the centrally acting antiobesity agent, namely, Sibutramine (Reductil), which produces unwanted side effects such as trouble sleeping, constipation, and dry mouth as well as increased heartbeat, increased blood pressure, awareness of the heartbeat (palpitations), headache, anxiety, or dizziness [8–10]. Consequently, the Food and Drug Administration (FDA) of the USA withdrew Sibutramine in October 2010.

Surgical procedures such as gastric bypass operations are generally reserved for people with morbid obesity (BMI > 40) who instituted but failed an adequate exercise and diet program (with or without adjunctive drug therapy) or patients presenting with comorbid conditions such as hypertension, impaired glucose tolerance, diabetes mellitus, hyperlipidaemia, and obstructive sleep apnoea [11].

Chinese herbal medicine has been used for weight management both in China and in western countries [12]. However, there is a lack of rigorously conducted randomized, controlled clinical trials published on Chinese herbal medicines for weight management in the international peer-reviewed scientific literature.

In 2004, Hioki et al. demonstrated the effectiveness and safety of a traditional Chinese formula (Bofutsusho-san) in obese Japanese women with impaired glucose tolerance. Findings from this study revealed significant improvement in both treatment and placebo groups compared to baseline. However, waist and hip circumference measurements of subjects in the Bofutsusho-san treatment group were also significantly improved compared to that of the control group [13].

A number of animal studies support the use of Chinese herbal medicine formulas for treating obesity and have shown other beneficial effects. For example, the Bofutsusho-san formula has been shown to prevent intimal thickening and vascular smooth muscle cell proliferation in rats [14]. Furthermore, an *in vivo* study demonstrated that a Chinese herbal formula significantly reduced the weight of overweight rats and suppressed their appetites [15]. A herbal treatment, known as 9D-ASR also leads to a decrease in the weight of overweight rats [16, 17]. Another herbal formula (PM-F2-OB) also demonstrated anti-obesity effects in overweight rats [18]. To evaluate the potential mechanism of actions of many Chinese herbs that are traditionally used for weight management, Tian et al. carried out investigations of 31 herbs on rats and found that 17 of them inhibited the enzyme, fatty acid synthase [19]. It was also interesting to note that some agents such as conjugated linoleic acid, catechins, and synephrine hydroxycitric acid often used for weight reduction were also found in Chinese herbal medicines [20, 21].

Until recently, widely used herbal supplements for weight loss contained ephedra alkaloids and herbal forms of caffeine, which are constituents of Mahuang [20]. Many clinical trials have been conducted to investigate the effectiveness of these two constituents for treating obesity [22, 23]. However, certain adverse effects associated with ephedrine and caffeine, such as central nervous system stimulation and increases in blood pressure and serum glucose, have been reported [20]. The use of ephedra can lead to life-threatening adverse cardiac effects [24], which include myocardial infarction, stroke, and death outside of a hospital [25]. Consequently, the sale of products containing ephedrine has been prohibited in the USA [26, 27]. There have also been reported cases of hepatotoxicity caused by Chinese dietary weight loss formulas containing fenfluramine and nitroso-fenfluramine. Kidney diseases or urothelial carcinoma related to the use of Chinese herbs containing aristolochic acids have also been reported [28]. RCM-104, the Chinese herbal formula used in the present study, contained none of these substances.

RCM-104 is an RMIT Chinese herbal medicine formula with three herbal ingredients that are commonly used in daily practice. These 3 herbs were carefully selected based on both Chinese medicine theory and existing published literature of basic research. A previous study on green tea extract had shown a 4.6% decrease in body weight and 4.5% decrease in waist circumference after 12 weeks, possibly via inhibition of intestinal lipases and stimulation of thermogenesis [29].

The aim of this study was to evaluate the efficacy and safety of RCM-104 through a rigorous double-blinded, randomized, placebo-controlled clinical trial.

## 2. Methods

### 2.1. Study Design

The trial was approved by the RMIT University Human Research Ethics Committee, and a Clinical Trial Notification (CTN) application was filed with the Therapeutic Goods Administration (TGA-2007/313), Department for Health and Ageing, Australian Federal Government, Canberra, Australia. The trial has been registered with Australian and New Zealand Clinical Trial Registry (ACTRN12607000255482).

Subjects and personnel who were involved in the clinical trial were blinded to the participant's group allocation. A pharmacist, responsible for prepacking and dispensing the RCM-104 and matched placebo capsules into identical packages, was the only person with access to the randomization allocation codes.

**Figure 1:**
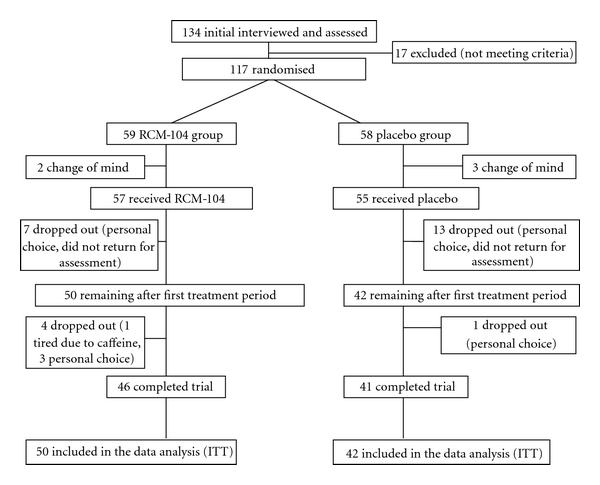
Clinical trial profile.

Subjects were given written information and a verbal explanation concerning the study prior to obtaining consent for their participation. After the two-week baseline assessment, the subjects were randomly assigned into either the RCM-104 treatment or placebo groups using treatment allocation codes generated by a statistician and designed to ensure balance of gender, age, and severity of obesity between groups (Figure 1).

Subjects were required to take either the RCM-104 or placebo capsules for 12 weeks with measures of BW, BMI, and body fat taken once every 4 weeks as well as at 12 weeks after intervention.

### 2.2. Subjects

Subjects' ages ranged from 18 to 60 years with a BMI ≥ 30 kg/m^2^. The exclusion criteria were (1) losing more than 5 kg in the past 3 months; (2) endocrine disorders other than type 2 diabetes mellitus; (3) uncontrolled hypertension; (4) autoimmune or cardiovascular diseases or carrying a pace-maker; (5) lactating or pregnant women; (6) those using drugs affecting the central nervous system or lipid lowering drugs; (7) obesity known to be caused by pharmacotherapy; (8) therapy for weight control in the last 6 months; (9) renal or hepatic disease; (10) unable to read or understand English. The subjects were not allowed to receive other obesity management and were asked to keep to their existing diet and life style during the study period. All subjects were free to withdraw at any time during the course of the study.

The study was advertised in the local newspapers and websites. Flyers advertising the study were displayed in local medical centers and university campuses. After a brief telephone screening interview, subjects were invited to a face-to-face interview to ensure they understood the aims of the trial and satisfied the inclusion and exclusion criteria.

### 2.3. Treatment

All subjects were instructed to take 4 capsules per time, three times per day. The treatment group received RCM-104 capsules containing 500 mg granule extract from dried herbals according to the pre-set standard procedures with certificate of analysis. The standard markers were Chrysophanol (0.0317 mg/g), Epicatechin (EC—10.89 mg/g), Caffeine (31.24 mg/g); Epigallocatechin gallate (EGCG—52.34 mg/g); Epicatechin gallate (ECG—13.63 mg/g) and Rutin (62.09 mg/g). The ingredients include *Camellia sinensis* (Lu Cha Ye: 40%), *Cassia obtusifolia* (Jue Ming Zi: 40%), *Sophora Japonica* (Huai Hua: 20%). These ingredients are listed in Australian Register of Therapeutic Goods (ARTG) as approved substances for human consumption by the Therapeutic Good Administration (TGA). The placebo group received herbal starch capsules that contained no active substances, with an identical appearance to the RCM-104 capsules. Both granules of RCM-104 and placebo were in standard capsules produced by Sun Ten Pharmaceuticals Co Ltd Taiwan that holds a TGA approved Good Manufacturing Practice (GMP) certificate. The trial medication compliance was monitored by counting left-over capsules to determine the number of capsules taken by subjects and checking on the completeness of the data on forms returned to the trial team.

### 2.4. Measurements

All anthropometric measurements were carried out using standardized methods and performed at the beginning of each 4 weekly visit. Height was measured with a wall-mounted stationmaster without wearing shoes to the nearest 0.1 cm; weight was measured while wearing light clothes without shoes on a calibrated balance beam scale to the nearest 0.1 kg. BMI was calculated according to the formula: BMI = body weight (BW)/squared height (kg/m^2^). Waist circumference (WC) was measured mid-way between the lateral lower rib margin and the iliac crest, and hip circumference (HC) was measured at the levels of the prominence of greater trochanters. Body fat (BF) composition was determined using a standard Bioimpedance Analyzer (BIA, Model HBF-522; Omron, Kyoto, Japan) which calculated lean mass and fat mass using an algorithm based on electrical resistance, weight, age, height, and gender.

The resting metabolic rate was determined by Fitmate (Biomedex Pty. Ltd. 1/1 Pioneer Drive, Bellambi, NSW, Australia). Subjects were instructed not to exercise or consume stimulants such as alcohol, tea, or coffee at least 2 hours prior to the test. Blood pressure and heart rate were measured three times in a resting seated position with the rest period of 1 minute between the measurements using ITO blood pressure monitor, and the average of all three measurements was recorded.

Blood collections for serological data were conducted only at the first and the last visit (week 12). The serological data included cholesterol, low density lipoprotein cholesterol (LDL), high-density lipoprotein (HDL) cholesterol, triglycerides, fasting insulin and glucose for calculation of insulin sensitivity (HOMA-IR), and renal and liver functions tests.

Self-assessment questionnaires were used to monitor quality of life using validated Weight-Related Symptom Measure (WRSM) and the Obesity & Weight-Loss Quality of Life measure (OWLQOL) questionnaires (University of Washington, 2004) prior to each visit.

### 2.5. Food Intake Records

Subjects were asked to maintain their normal diet and routine activities. They were instructed to record their diet in the dietary record form. The forms include day by day, meal by meal records of all food and liquids consumed over 3 days at the beginning of the trial and further random 3 days in each treatment period. The food intake records were subsequently analysed using a standard dietary software package (FoodWorks, Xyris, Brisbane, Australia) incorporating the latest Australian database of food composition (NUTTAB 2006, FSANZ, Canberra, Australia). Nutrient intake was averaged over the initial 3-day period to determine the daily intake of macro- and micronutrient, fat subtypes, and total energy as the subject's baseline diet. Dietary data collected during study periods were compared to baseline to determine any change in dietary intake (data not provided).

### 2.6. Statistical Analysis

All data were analysed using the Statistical Package for the Social Sciences (*SPSS*, version 18 for Windows). Intention to treat (ITT) analysis included all randomized patients with baseline data with at least one outcome after the interventions were determined using the last-value-carried-forward method. Outcomes were evaluated by comparing baseline data with data after 4 weeks of intervention for the variables BW, BMI, WC, HC, Hip to waist ratio (HWR), and BF.

The data from nonrepeated measures including blood tests were analyzed using *t*-tests. Ordinal data variables involving baseline and end of study within each treatment group were analyzed using the Wilcoxon signed-rank test. Gender balance in the two groups was assessed using the Chi-square test. *ANCOVA* was used to assess whether the treatment and placebo groups were significantly different on variables assessed at the end of the study using baseline as covariate. All statistical tests were assessed at **α** = 0.05 with adjustments to **α** where necessary to accommodate testing involving multiple outcomes.

## 3. Results

### 3.1. Baseline Subject Characteristics

Of 133 obese subjects screened, 117 fulfilled the inclusion criteria and were randomized into placebo (*n* = 59) and treatment groups (*n* = 58, Table 1).

Thirteen (13) subjects of the treatment group and 17 subjects of the placebo group prematurely withdrew before the commencement of the treatment phase or did not return for the assessment after the first treatment period due to personal and other reasons. Ninety-two subjects were included in an ITT analysis. No subjects were removed or withdrew from the study due to serious adverse events (Figure 1).

### 3.2. Treatment Effects

#### 3.2.1. Body Weight, BMI, and Body Fat Composition

**Figure 2:**
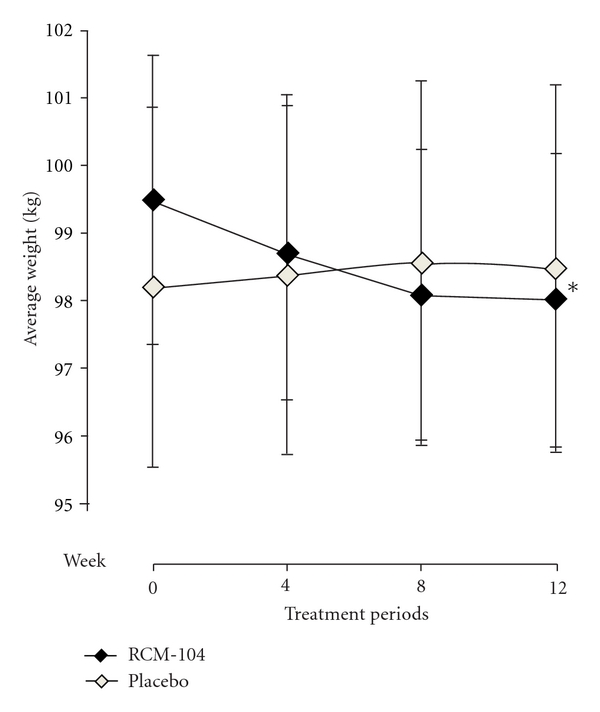
Average weights (Kg) after 12-week treatment. The plotted line graphs indicate the means ± SD of change of weight at each assessment visit, RCM-104 group (*◆*) and placebo control group (*◊*). (∗) indicates value that is significantly different from that of placebo group using ANCOVA and ITT, (*P* = 0.006).

**Figure 3:**
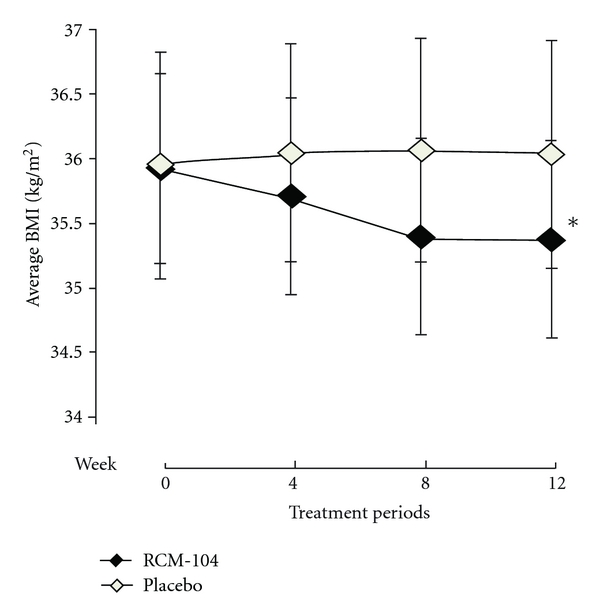
Average body weight index (Kg/m^2^) after 12-week treatment. The plotted line graphs indicate the means ± SD of change of BMI at each assessment visit, RCM-104 group (*◆*) and placebo control group (*◊*). (*) indicates the values significantly different than that of placebo group using *ANCOVA* and ITT, (*P* = 0.027).

At baseline, the placebo group had a mean weight (98.2 ± 17.3 kg) and mean BMI (36.0 ± 5.5 kg/m^2^) similar to those in the treatment group (99.5 ± 15.1 kg), (35.3 ± 4.8), respectively. *ANCOVA *was used for assessing treatment outcomes in weight and BMI between the two groups after 12 weeks of treatment using the baseline as a covariate. When both groups were compared after 12 weeks of treatment with the baseline as covariate, there was a significant difference between the two groups on weight (*P* = 0.006, Figure 2) and BMI (*P* = 0.027,Figure 3). Note that we used Hochberg's step-up procedure to control Type I error to assess statistical significance. In doing so, we included BW, BMI, and BF composition as the three core variables that were used to assess the efficacy of the treatment. Within the treatment group, the reduction in weight and BMI were statistically significant.

**Figure 4:**
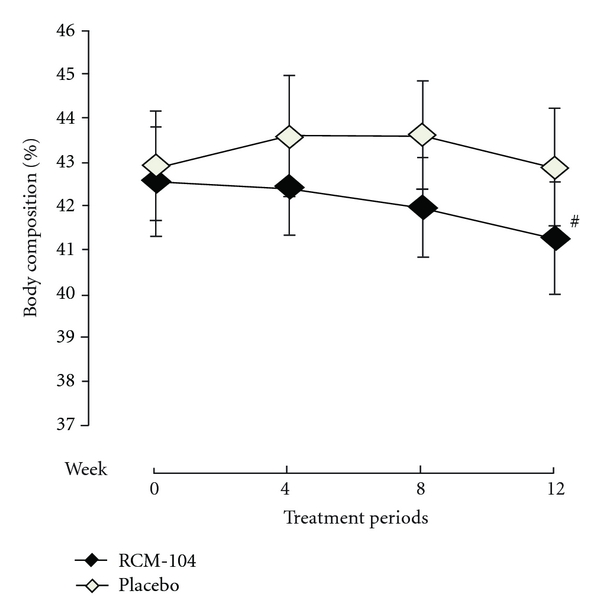
Change in body fat composition (%) after 12-week treatment. The plotted line graphs indicate the means ± SD of change of body fat composition at each assessment visit, RCM-104 group (*◆*) and placebo control group (*◊*). (^#^) indicates value not significantly different using *ANCOVA* and ITT, (*P* > 0.05).

The analysis showed that after 12 weeks of treatment, there was no significant reduction in the BF composition between the RCM-104 group and the placebo group (*P* = 0.151, Figure 4).

#### 3.2.2. Quality of Life

At baseline, there were no statistically significant differences in any of the 20 quality of life measures (Weight-Related Symptoms and How Much They Bother You) between the two groups. The RCM-104 group showed significant improvement compared to baseline on only two items of the quality of life measure, which assessed shortness of breath and physical stamina (*P* = 0.001), while the placebo group showed no significant improvement on any of the items after controlling for Type I error due to multiple testing via the Holm's step-down procedure (Table 2). (The smallest *P*-value was compared against **α**/*m*, the next smallest against *α*/(*m* − 1), etc., where *α* = 0.05 and *m* = 20 variables. Note that we have only included 5 of the 20 variables in Table 2 on the basis of having the smallest *P*-values. All other variables had associated *P*-values, which were large enough not to have reached significance.) When the RCM-104 group was compared to the placebo group after 12 weeks of treatment, an *ANCOVA* analysis with baseline as the covariate, and using the Holm step-down procedure, as described above, for controlling Type I error, the only quality of life measure that showed significant difference in symptoms between the two groups was shortness of breath (*P* = 0.002) with the RCM-104 group showing significant reduction in symptoms (*P* = 0.001, Table 2).

Similarly, after 12 weeks, eleven items of Weight-Loss Quality of Life (your feelings about your weight—Table 3) were significantly improved in the treatment group, while only 2 items were significantly improved in the placebo group. When the RCM-104 group was compared to the placebo group after 12 weeks of treatment, an *ANCOVA* analysis with baseline as the covariate, and using the Holm's step-down procedure for controlling Type I error, there were no statistically significant differences between the two groups on all of the 17 items except one (“I get discouraged when I try to lose weight”) on the Weight-Loss Quality of life Scale. (Here we used *α* = 0.05/17 = 0.0029 for assessing the smallest *P*-value (Table 3)).

#### 3.2.3. Resting Metabolic Rate (RMR) and Food Intake

Both within group and between the groups analyses did not reveal significant changes in RMR (Table 4) for both groups.

There were some changes in the food intake by the subjects during the trial. However, the changes were not significant (data will be presented in a future journal article).

#### 3.2.4. Laboratory Data

There were no significant differences between the two groups with respect to renal and liver function tests before and after the treatment period. Similarly, there were no statistically significant differences in the serological data between the 2 groups.

#### 3.2.5. Medication Compliance

Overall, the compliance of medication was 88% with 88.6% in the placebo and 87% in the treatment group.

#### 3.2.6. Self-Reported Undesired Symptoms

Within the RCM-104 group, there were reports of nausea (4 cases) and headache (9 cases) while there were 2 reports of decrease in appetite in the placebo group. Both of these symptoms occurred during the first treatment period and none of these were serious. No subject withdrew from the study due to adverse events.

## 4. Discussion

This study was designed to evaluate the effect of a Chinese herbal medicine formula RCM-104 in the treatment of simple obesity. There was a high level of willingness to participate in this study as all 117 subjects who satisfied inclusion criteria gave informed consent to participate in the trial.

The results of the present study showed statistically significant differences in BW and BMI between the participants of RCM-104 and placebo groups after 12 weeks of treatment and significant improvements in quality of life of participants in the RCM-104 group.

Because obese individuals often have other associated health issues, guidelines for obesity management prefer modest and safe weight loss and good weight maintenance to target an ideal weight [31].

The RCM-104 is composed of 3 herbs: Camellia Sinensis (Lu Cha Ye—Green tea), Semen Cassiae (Jue Ming Zi), and Flos Sophorae (Huai Hua). While the mechanism of actions of this formula is unknown, a study has shown that green tea extract stimulated thermogenesis and fat oxidation potentially may influence body weight and body composition by increasing energy expenditure [32].

It has been suggested that synergistic interactions between catechin-polyphenol and caffeine augment and prolong sympathetic stimulation of thermogenesis and that this may play an important role in the management of obesity [32]. Thus the actions of green tea may contribute to reducing body fat as previous experimental and clinical studies have reported that green tea extract, which is rich in catechins and caffeine, is an effective potentiator of sympathetically mediated thermogenesis [33]. Further actions of green tea include EGCG's inhibition of the enzyme catechol-*O*-methyltransferase, which prolongs the action of catecholic compound on energy expenditure [33]. Studies also suggested that EGCG stimulates thermogenesis, with caffeine enhancing this action. Other studies have found that catechin polyphenols and caffeine increase 24-hour energy expenditure and fat oxidation in humans, which contribute to weight reduction [33]. This present study did find some increase in RMR; however, the difference between the two groups was not significant.

In Chinese medicine practice, Jue Ming Zi is known to clear heat, moistens and lubricates the intestines, and therefore acts as a mild laxative [34]. The anti-obesity effects of this herb, however, may be attributed to the inhibition of fatty acid synthase [19], which is shown in the reduction of body fat composition. Traditionally, Huaihua is used to cool blood and stop bleeding which is an anti-inflammatory effect. The anti-obesity effect of Huaihua is attributed to its diuretic and laxative actions [35].

In human studies, green tea has been found to significantly increase energy expenditure, lower body weight, and decrease waist circumference without changing heart rate or blood pressure [33]. Obesity is associated with many other health problems [2] and even small reductions in weight can lead to significant improvements in quality of life of obese individuals [36]. In this study, we found that RCM-104 significantly improved many aspects of quality of life consistent with Chinese medicine diagnosis theory. The reduction of joint pain among the participants in this study may be due the anti-inflammatory action of Huaihua and Juemingzi [35]. The cholesterol levels have been unchanged, which is not consistent with an animal study by [37] that Huaihua lowered hepatic and blood cholesterol levels, but may be due to a relatively small proportion of Huaihua in RCM-104. Chronic obstructive pulmonary disease (COPD) often coexists within the obese population [38]. In this study, subjects in the treatment group showed significant improvement of shortness of breath symptoms. This effect maybe due to the antioxidant effects of green tea catechins [39].

There has been general concern of the lack of safety of specific Chinese herbal medicine. Kidney failure due to herbal weight loss pills has been reported [40]. The results of this study have shown only mild undesirable adverse events such as headache and nausea that occurred during the first treatment period. This is consistent with reports from previous studies [41, 42]. A study by Pisters et al. in 2001 showed that a single oral consumption of 800 mg epigallocatechin gallate (EGCG) might cause mild headache [43, 44]. A study by Hsu et al. 2008 did not detect EGCG in the serum sample of subjects at the dosage of 302 mg of EGCG per day. In the present study, RCM-104 contained 314.04 mg of EGCG in the daily dosage. It has been previously reported that green tea could cause slight gastrointestinal disturbances such as nausea [45]. Although Juemingzi is included in the list of poisonous plants of North Carolina, Russell et al. stated that the toxic effects are only associated with the intake of large quantities without specifying dosage [46]. However, an animal study has shown no changed serum aspartate aminotransferase, creatine phosphokinase, and lactic dehydrogenase activity at the dosage of 1.19% of body weight per day [47]. Another animal study did not see any sign of chronic ingestion when consuming 0.15% Juemingzi of the diet, and Intermittent mild diarrhea was observed in animals consuming high doses (5%) of Juemingzi in their diet [48]. Juemingzi has been safely used in Chinese medicine practice for hundreds of years. In this study, we used 2.4 g of Juemingzi extract, which is less than half of recommended dosage of 5 g extract per day [35]. Huaihua has been used in many Asian countries as daily tea. In daily practice, overuse of Huaihua can cause mild diarrhea in some cases [35]. The recommended safe dosage for this herb is 5 g [34] of extract per day, but only 1.2 g per day was used in this study. Based on the dosages used in this study and the findings, RCM-104 appeared to be safe and well tolerated by the subjects.

## 5. Conclusion

RCM-104 is well tolerated and appears to be effective in reducing weight and improving quality of life in obese individuals after 12 weeks. Long-term follow-up studies in larger populations are required to determine if weight loss is sustained.

## Figures and Tables

**Table 1:** Demographic and baseline health characteristics of all randomized subjects by group.

	RCM-104 (*n* = 59, Mean ± SD)	Control (*n* = 58, Mean ± SD)	*P-*value
Gender			
Male	10	10	0.97
Female	49	48	
Age (years)	39.3 ± 13.2	40.4 ± 10.2	0.61
Weight (kg)	99.5 ± 15.1	98.2 ± 17.3	0.67
Male	105.5 ± 15.1	108.3 ± 21.1	0.41
Female	98 ± 14.7	96.8 ± 16.1	0.67
BMI (kg/m^2^)	35.3 ± 4.8	36.0 ± 5.5	0.47
Heart rate (beats/min)	72.6 ± 9.2	73 ± 11.4	0.84
Systolic blood pressure (mmHg)	122.8 ± 15.5	126.2 ± 19.8	0.30
Diastolic blood pressure (mmHg)	80.8 ± 10.8	82.9 ± 13.7	0.16
HbA1c (%)	5.3 ± 0.3	5.6 ± 0.8	0.008
Triglyceride (mmol/L)	1.5 ± 0.8	1.6 ± 0.8	0.50
Total Cholesterol (mmol/L)	5.6 ± 1.5	5.5 ± 1.5	0.71
LDL Cholesterol (mmol/L)	3.6 ± 0.8	3.6 ± 1.5	1.00
HDL Cholesterol (mmol/L)	1.5 ± 0.08	1.4 ± 0.23	0.002
Insulin (mIU/L)	10.7 ± 9.2	15.1 ± 3.6	0.001
Glucose (mmol/L)	4.8 ± 0.5	5.2 ± 1.5	0.058
Homa-index (%)	2.3 ± 2.2	3.9 ± 1.0	<0.001

**Table 2:** Weight-related symptoms and how much they bother you.

Symptoms	Study period	RCM-104 (*n* = 48)^#^ value ± SD	Placebo (*n* = 42) value ± SD	*P* value**
Shortness of breath	Baseline	1.2 ± 1.27	1.1 ± 1.23	
	Final	0.6 ± 0.92	1.2 ± 1.21	
	*P* value*	**0.001**	0.760	**0.002**

Sensitivity to cold	Baseline	1.0 ± 1.57	0.8 ± 1.12	
	Final	0.5 ± 0.92	0.9 ± 1.23	
	*P* value*	0.008	0.628	0.011

Leakage of urine	Baseline	0.4 ± 1.09	0.4 ± 0.73	
	Final	0.4 ± 0.91	0.8 ± 0.93	
	*P* value*	0.521	0.005	0.005

Increased sweating	Baseline	0.9 ± 1.56	0.7 ± 1.20	
	Final	0.5 ± 1.11	0.8 ± 1.15	
	*P* value*	0.030	0.547	0.057

Decreased physical stamina	Baseline	1.6 ± 1.91	1.2 ± 1.54	
	Final	0.7 ± 1.23	1.1 ± 1.53	
	*P* value*	**0.001**	0.660	0.049

Joint pain	Baseline	1.5 ± 1.62	1.8 ± 1.83	
	Final	0.8 ± 1.18	1.6 ± 1.56	
	*P* value*	0.003	0.748	0.007

Weight-Related Symptom Measure (WRSM) is 7-point scaled: 0 = not at all; 1 = hardly; 2 = somewhat; 3 = moderately; 4 = a good deal; 5 = a great deal; 6 = a very great deal.

*Comparison within the group between baseline and final visits using Wilcoxon-signed rank test.

**Comparison between groups at the end of trial.

For the above, the *P* values were assessed using Holm's step-down procedure, with *α* = 0.05/20 = 0.0025 for assessing the smallest *P* value, *α* = 0.05/19 = 0.0026 for the next smallest *P* value, and so forth. Significant *P* values are indicated in bold [30]. Note: only results for symptoms showing small *P* values are displayed.

^#^Sample size for treatment group reduced from 50 to 48 as Quality of Life data were unavailable for two participants.

**Table 3:** Obesity and weight-loss quality of life-your feelings about your weight.

Symptoms	Study period	RCM-104 (*n* = 48)^#^ value ± SD	Placebo (*n* = 42) value ± SD	*P* value**
Because of my weight, I try to wear clothes that hide my shape	Baseline	4.0 ± 1.57	3.9 ± 1.74	
	Final	3.0 ± 1.94	3,5 ± 1.67	
	*P* value*	**<0.001**	0.033	0.021

I feel frustrated that I have less energy because of my weight	Baseline	3.5 ± 1.91	3.2 ± 1,77	
	Final	2.54 ± 1.97	2.8 ± 1.61	
	*P* value*	**<0.001**	0.058	0.025

I feel guilty when I eat because of my weight	Baseline	3.3 ± 1.80	3.0 ± 1.75	
	Final	2.5 ± 01.94	2.7 ± 1.76	
	*P* value*	**<0.001**	0.025	0.093

I am bothered about what other people say about my weight	Baseline	2.8 ± 2.06	2.9 ± 1.75	
	Final	2.21 ± 1.99	2.5 ± 1.77	
	*P* value*	0.033	0.047	0.590

Because of my weight, I try to avoid having my photograph taken	Baseline	3.1 ± 2.17	3.4 ± 2.07	
	Final	2.35 ± 2.09	2.95 ± 1.94	
	*P* value*	**0.002**	0.016	0.374

My weight prevents me from doing what I want to do	Baseline	2.7 ± 2.06	2.9 ± 1.73	
	Final	2.2 ± 1.86	2.3 ± 1.65	
	*P* value*	0.045	**0.001**	0.608

I worry about the physical stress that my weight puts on my body	Baseline	3.6 ± 1.86	3.9 ± 1.78	
	Final	2.8 ± 1.83	3.4 ± 1.71	
	*P* value*	**0.003**	0.028	0.329

I feel depressed because of my weight	Baseline	2.9 ± 1.92	2.8 ± 1.74	
	Final	2.3 ± 2.02	2.6 ± 1.78	
	*P* value*	**0.001**	0.148	0.106

I feel ugly because of my weight	Baseline	3.1 ± 2.05	3.1 ± 1.84	
	Final	2.4 ± 2.19	2.6 ± 1.97	
	*P* value*	**<0.001**	**0.002**	0.072

I worry about the future because of my weight	Baseline	3.6 ± 1.89	3.4 ± 1.68	
	Final	2.5 ± 1.82	3.2 ± 1.60	
	*P* value*	**<0.001**	0.231	0.702

I envy people who are thin	Baseline	2.8 ± 2.12	2.6 ± 1.96	
	Final	2.2 ± 2.08	2.4 ± 2.08	
	*P* value*	**<0.001**	0.382	0.267

I feel that people stare at me because of my weight	Baseline	2.3 ± 1.99	1.8 ± 1.79	
	Final	1.8 ± 1.95	1.5 ± 1.66	
	*P* value*	0.014	0.254	0.332

I have difficulty accepting my body because of my weight	Baseline	3.0 ± 2.13	2.9 ± 1.93	
	Final	2.3 ± 2.05	2.6 ± 1.90	
	*P* value*	0.006	0.170	0.179

I am afraid that I will gain back any weight that I lose	Baseline	3.8 ± 1.91	3.7 ± 1.72	
	Final	2.9 ± 2.13	3.3 ± 1.97	
	*P* value*	**0.002**	0.019	0.149

I get discouraged when I try to lose weight	Baseline	3.7 ± 1.92	3.1 ± 2.03	
	Final	2.7 ± 2.18	3.1 ± 1.99	
	*P* value*	**<0.001**	0.848	**0.002**

Obesity & Weight-Loss Quality of Life measure (OWLQOL) questionnaires are 7-point scaled: 0 = not at all; 1 = hardly; 2 = somewhat; 3 = moderately; 4 = a good deal; 5 = a great deal; 6 = a very great deal.

*Comparison within the group between baseline and final visits using Wilcoxon signed-rank test.

**Comparison between groups at the end of trial.

For both the above, the *P* values were assessed using Holm's step-down procedure, with *α* = 0.05/17 = 0.0029 for assessing the smallest *P* value, **α** = 0.05/16 = 0.0031 for the next smallest *P* value, and so forth. Significant *P* values are indicated in bold.

Note: only symptoms showing small *P* values are displayed.

^#^Sample size for treatment group reduced from 50 to 48 as Quality of Life data were unavailable for two participants.

**Table 4:** Changes in anthropometric parameters of RCM-104 and placebo groups.

	Study period	RCM-104 (*n* = 50) value ± SD	Placebo (*n* = 42) value ± SD	*P* value**
Body weight (kg)	Baseline	99.5 ± 15.1	98.2 ± 17.3	
	Final	98.0 ± 15.4	98.7 ± 17.5	
	*P* value*	**0.002**	0.510	**0.006**

Body mass index (kg/m^2^)	Baseline	35.9 ± 4.9	35.9 ± 5.9	
	Final	35.3 ± 5.7	36.1 ± 5.9	
	*P* value*	**0.008**	0.676	0.027

Body fat (%)	Baseline	42.2 ± 9.2	42.9 ± 8.4	
	Final	41.3 ± 9.2	43.0 ± 7.8	
	*P* value*	0.104	0.935	0.151

Waist circumference (cm)	Baseline	108.5 ± 12.3	107.4 ± 11.04	
	Final	106.6 ± 12.5	106.3 ± 12.3	
	*P* value*	0.014	0.182	0.445

Hip circumference (cm)	Baseline	124.4 ± 12.3	124.7 ± 12.5	
	Final	122.9 ± 12.0	125.1 ± 12.8	
	*P* value*	0.034	0.544	0.042

Waist to Hip Ratio	Baseline	0.87 ± 0.08	0.87 ± 0.08	
	Final	0.87 ± 0.07	0.85 ± 0.081	
	*P* value*	0.382	0.067	0.356

Systolic Blood pressure (mmHg)	Baseline	122.8 ± 14.1	126.2 ± 16.8	
	Final	117.8 ± 14.0	118.3 ± 15.6	
	*P* value*	0.738	0.854	0.685

Diastolic blood pressure (mmHg)	Baseline	80.8 ± 9.9	82.9 ± 11.7	
	Final	81.1 ± 9.8	81.8 ± 10.4	
	*P* value*	0.853	0.855	0.934

Heart rate (beats/min)	Baseline	72.6 ± 8.5	73.1 ± 9.7	
	Final	74.6 ± 9.2	73.2 ± 9.1	
	*P* value*	0.084	0.897	0.281

RMR (Kcal/day)	Baseline	1353.8 ± 353	1333.3 ± 315	
	Final	1478.9 ± 330	1450.6 ± 276	
	*P* value*	0.085	0.019	0.827

*Comparison within the group between baseline and final visits.

**Comparison between groups at the end of trial.

(*ANCOVA*, using baselines as covariate). Body Weight, Body Mass Index, and Body Fat were used as a single family of variables when assessing significance using Hochberg's Step-up procedure for adjusting significance levels as these variables were the focus of the study.
